# Selection of a novel DNA aptamer against OFA/iLRP for targeted delivery of doxorubicin to AML cells

**DOI:** 10.1038/s41598-019-43910-3

**Published:** 2019-05-14

**Authors:** Yacong An, Yan Hu, Xundou Li, Zhaoyi Li, Jinhong Duan, Xian-Da Yang

**Affiliations:** Institute of Basic Medical Sciences, Chinese Academy of Medical Sciences & Peking Union Medical College, Beijing, 100005 China

**Keywords:** Acute myeloid leukaemia, Targeted therapies

## Abstract

The standard treatment for most acute myeloid leukemia (AML) is chemotherapy, which is often associated with severe adverse effects. One strategy to reduce the adverse effects is targeted therapy that can selectively deliver anticancer drugs to tumor cells. Immature laminin receptor protein (OFA/iLRP) is a potential target for AML treatment, because it is over-expressed on the surface of AML cells but under-expressed in normal tissue. In this study, we developed the first aptamer for OFA/iLRP and explored its potential as a targeting ligand for delivery of doxorubicin (Dox) to AML cells *in vitro*. The selected aptamer (AB3) was a 59-base DNA oligonucleotides. It bound to OFA/iLRP structure with a K_d_ of 101 nM and had minimal cross-reactivity to albumin, trypsin, or ovalbumin. Moreover, AB3 could bind to OFA/iLRP-positive AML cells but not the OFA/iLRP-negative control cells. An aptamer-doxorubicin (Apt-Dox) complex was formed by intercalating doxorubicin into the DNA structure of AB3. Apt-Dox selectively delivered Dox to OFA/iLRP-positive AML cells but notably decreased the drug intake by OFA/iLRP-negative control cells. In addition, cytotoxicity study revealed that Apt-Dox efficaciously destroyed the OFA/iLRP-positive AML cells, but significantly reduced the damage to control cells. The results indicate that the OFA/iLRP aptamer AB3 may have application potential in targeted therapy against AML.

## Introduction

Acute myeloid leukemia (AML) is a serious threat to human health and accounts for about 147 thousands deaths globally in 2015^1^. It is characterized by the rapid growth of immature myeloid precursors in bone marrow and blood^2^. Despite the progress in biomedical sciences, AML still has a relatively poor clinical outcome. Among leukemic diseases that cause mortality in United States, AML is the most common type, with a 5-year survival of only 30–40%^2,3^. The standard treatment for most AML at present is chemotherapy, which is often associated with severe side effects. Induction chemotherapy with cytarabine and anthracycline followed by consolidation chemotherapy has remained unchanged as the standard regimen for more than 3 decades^4^. In addition to killing leukemic cells, however, chemotherapeutics are also highly toxic to normal cells, causing serious adverse effects. Additionally, more than half of AML patients at diagnosis are older than 65 years, and frequently have antecedent hematologic disorders, adverse cytogenetic abnormalities, and other clinically significant comorbid conditions^3^. Consequently, aged patients often have poor tolerance to cytotoxic drugs with enhanced adverse effects^5^, and the median unadjusted overall survival of patients over 66 years is less than one year^6^. At present, there is an urgent need to develop novel AML therapeutic strategies that have decreased toxicity and improved efficacy.

Targeted therapy is a promising strategy for cancer treatment. By selective delivering chemotherapeutics to cancer cells rather than normal tissues, the undesirable side effects of conventional chemotherapy can be reduced, while the therapeutic efficacy is improved. It has been demonstrated that trastuzumab emtansine (T-DM1), an antibody-drug conjugate targeting human epidermal growth factor receptor 2 (HER2), prolonged progression-free survival (PFS) and overall survival (OS) compared with other anti-HER2 therapies in patients with HER2-positive metastatic breast cancer^7^. These studies indicate that targeted therapy has the potential to improve clinical outcome. For targeted AML treatment, gemtuzumab ozogamicin, a conjugation of anti-CD33 antibody and calicheamicin, was approved by FDA to treat patients with CD33-positive AML^8,9^. Ideally, the molecular target employed in targeted therapy is tumor specific. It should be highly expressed in tumor but under-expressed in normal tissue, in order to generate a disparity in the amount of drug delivered to tumor vs. normal tissue. CD33, however, is expressed on both the normal myeloid cells and the leukemic cells. This drawback sometimes limited the application of gemtuzumab ozogamicin in clinical settings due to side effects^10^. For AML treatment, therefore, it is necessary to explore new targets that are highly expressed on AML cells but under-expressed in normal tissue.

Oncofetal antigen/immature laminin receptor protein (OFA/iLRP) is a potentially important molecular target for treatment of AML and other malignancies. OFA/iLRP is a 37 kD membrane protein that has been detected on the cell surface of various malignant tumors, but not on the surface of normal cells. OFA/iLRP is expressed in AML, colon cancer, fibrosarcoma, ovarian cancer, lung cancer, and cervical cancer^11–15^. In normal tissues, OFA/iLRP is only expressed in embryonic and early fetal period, and disappears in later stages of development^16^. Barsoum *et al*. found that immunization with recombinant OFA/iLRP generated a significant antitumor effect in mice^17^, indicating that OFA/iLRP could be employed as a tumor-associated antigen in immunotherapy. Moreover, Scheiman *et al*. reported that treating tumor-bearing mice with short hairpin RNA (shRNA) against OFA/iLRP significantly inhibited tumor growth, suggesting that OFA/iLRP may be a potential target for gene therapy^18^. Importantly, OFA/iLRP expression is not only detected in many AML cell lines, but also in all clinical samples from AML patients^11^. Furthermore, OFA/iLRP-specific cytotoxic T lymphocytes (CTLs) could lyse leukemic cells from patients with AML^11^. These findings indicate that OFA/iLRP may potentially serve as an attractive molecular target for AML treatment. As mentioned above, OFA/iLRP has a molecular weight of about 37 kD, and is only expressed in embryonic or tumor cells. It should be noted that in mature tissues, two 37 kD OFA/iLRP molecules can form a dimer, generating a 67 kD protein named 67 LR or mature LRP^19^. The 67 LR is mainly localized in the cell membrane, and functions as a receptor for laminin^20^. It also serves as receptors for viruses, bacteria, and prions at times^21–23^. Numerous studies have shown that 67 LR is overexpressed in cancerous cells vs. their normal counterparts^24^. The overexpression of 67 LR is associated with metastatic aggressiveness in many malignancies, including cancers of breast, lung, ovary, prostate, stomach and thyroid, as well as leukemia and lymphoma^15,25–31^. Importantly, the expression of 67 LR in AML cells is significantly higher than that in normal myeloid cells^32^. Moreover, it has been reported that 67 LR expression shifted the characteristics of AML cells toward aggressive phenotype^33^. Since both OFA/iLRP and its dimer 67 LR are overexpressed in AML cells, targeting OFA/iLRP appears a reasonable strategy for AML treatment.

Tumor-targeted therapy requires ligands that can bind specifically to tumor markers. In addition to antibodies, aptamers may also serve as tumor-targeting ligands. Aptamers are short oligonucleotides that can form complicated 3D structures with target-binding properties similar to antibodies^34^. Moreover, aptamers have certain advantages as tumor-homing ligands, including low immunogenicity, better tumor penetration, easy chemical modification, and low production cost^35^. Numerous studies have shown that aptamers can be used as ligands in chemical analyses and diagnostic applications^36–38^. Aptamers can also be used as tumor-targeting ligands for delivery of doxorubicin or docetaxel to cancer cells^39–41^. An aptamer drug (Macugen) has been approved by FDA for treatment of age-related macular degeneration. Other aptamer-based drugs, including E10030^42^, NOX-A12^43^, and Pegpleranib^44^, are being investigated in clinical trials. These facts indicate that aptamers have application potential in drug development.

To date, no aptamers against OFA/iLRP have been reported in literature. In this study, we developed the first DNA aptamer against OFA/iLRP and evaluated its binding affinity and specificity. Because doxorubicin is one of the most widely used chemotherapeutic agents, we also constructed an aptamer-doxorubicin complex (Apt-Dox) as an AML-targeted drug delivery system. We now report that Apt-Dox can selectively deliver doxorubicin to AML cells *in vitro*.

## Results

### Selection of aptamer against OFA/iLRP

In this study, a peptide from the extracellular domain of OFA/iLRP with the sequence of NQIQAAFREPR was chosen to be the target^45^. This peptide represents an epitope that is exposed on the surface of the OFA/iLRP protein, according to studies of the crystal structure of the protein^46^. Utilizing an epitope peptide as the target for aptamer selection has several advantages. First, synthetic peptides of high purity can be obtained in sufficient quantity, facilitating the process of aptamer selection. Second, the epitope is exposed on the surface of the protein, and thus may serve as a better target for aptamers. Prior studies have shown that aptamers with decent functionality can be developed using synthetic peptides as the selection target. Ferreira *et al*. selected an aptamer with a peptide representing the immunodominant region of MUC1. This aptamer has been shown capable of binding with multiple MUC1-expressing cancer cell lines^47^. Liu *et al*. developed an aptamer using a peptide from the extracellular domain of HER2^40^. This HER2 aptamer could recognize HER2-expressing breast cancer cells in clinical samples with similar efficiency and specificity as the FDA approved staining kit^48^. Thus, it is feasible to employ a synthetic peptide as the target for aptamer selection.

**Figure 1:**
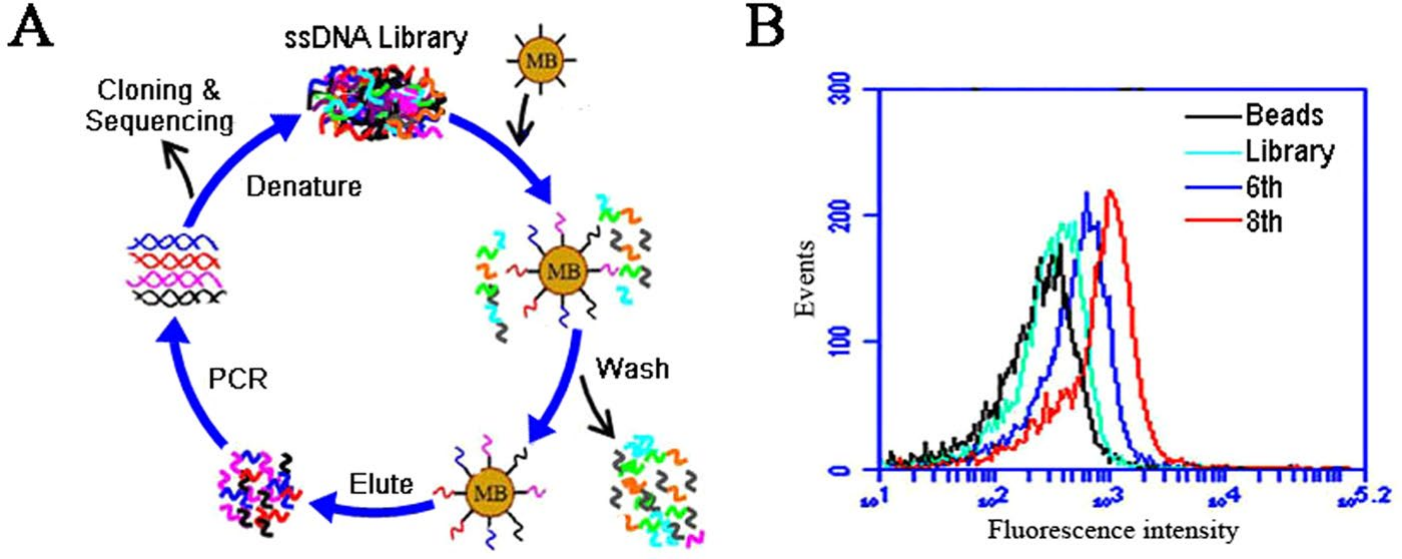
The process of aptamer selection. (**A**) Scheme of antigen based SELEX. The OFA/iLRP peptide-coated beads were incubated with ssDNA library, and washed to remove the unbound ssDNA. After heating at 95 °C for 5 min, the ssDNA still bound to the beads was eluted and amplified by PCR for next round of selection. In the last round of selection, the enriched target-binding ssDNA pool was cloned and sequenced to identify prospective OFA/iLRP aptamers. (**B**) Binding of the enriched ssDNA from the 6th and 8th round pools to beads coated with OFA/iLRP-peptide, as monitored by flow cytometry. The initial ssDNA library was used as the control.

The SELEX process is illustrated in Fig. 1A. The target peptide was conjugated covalently to the surface of magnetic beads. To select target-binding aptamers, the peptide-coated beads were incubated with a large variety of ssDNAs, and washed to remove the unbound DNA. The bead-bound DNA was eluted and amplified by PCR. The amplified dsDNA was separated into ssDNA which was used in the next round of selection. Flow cytometry was used to monitor the enrichment of OFA/iLRP aptamers. Compared with the initial ssDNA library, the binding to peptide-coated beads by selected DNA gradually strengthened with increasing rounds of selection (Fig. 1B). After eight rounds of selection, the enriched aptamer pool was cloned and analyzed. Among 120 clones, the aptamer AB3 showed relatively high capacity of binding to the OFA/iLRP-peptide. The sequence of this aptamer is 5′-TGCGTGTGTAGTGTGTCTGTTGTTTGTATTGTTGTCTATCCTCTTAGGGA TTTGGGCGG-3′.

### Characteristics of the aptamer

**Figure 2:**
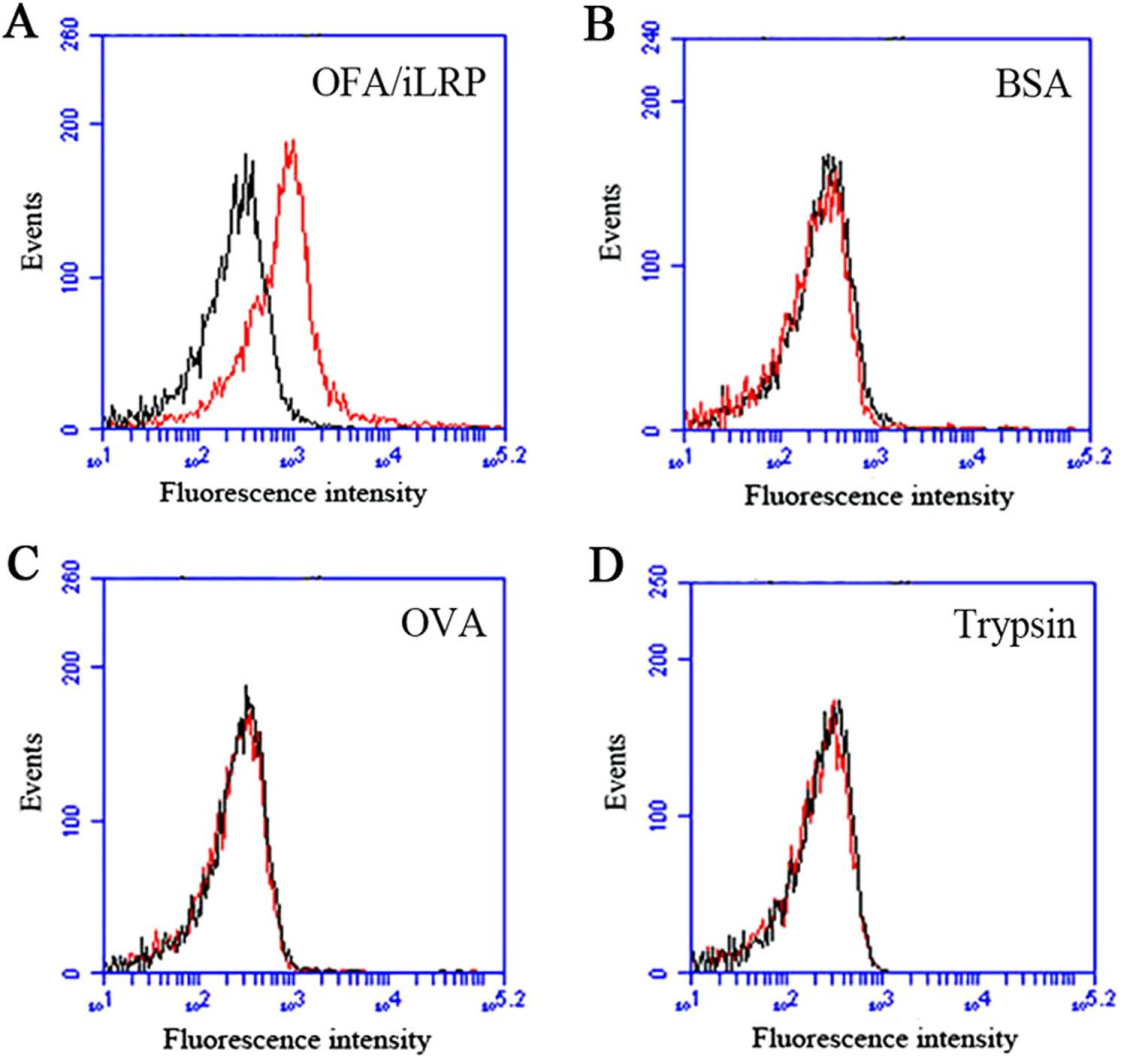
Binding properties of the AB3 aptamer. The bindings of FAM-labeled aptamer to OFA/iLRP-peptide (**A**), BSA (**B**), OVA (**C**), or trypsin (**D**) coated beads were analyzed by flow cytometry. The red curves represented the fluorescent signal of the aptamer AB3. The black curves represented the signal of initial ssDNA library, which served as the control here.

Binding specificity is one of the most essential characteristics of an aptamer, and often evaluated by comparing the aptamer’s bindings to the target molecule vs. several other control proteins. Albumin was often used as the control protein, because it is the most abundant protein in blood. Additionally, trypsin and OVA were also frequently employed as control proteins to test the binding specificity of the aptamers^40^. To evaluate the binding specificity of the selected aptamer AB3, beads coated with OFA/iLRP-peptide, BSA, OVA, or trypsin were incubated with FAM-labeled aptamer, washed, and evaluated by flow cytometry. As presented in Fig. 2, aptamer AB3 showed a strong binding to the OFA/iLRP coated beads (Fig. 2A), whereas the bindings to BSA (Fig. 2B), OVA (Fig. 2C), or trypsin (Fig. 2D) coated beads were very weak. The results indicated that aptamer AB3 had a targeting specificity towards OFA/iLRP structure.

**Figure 3:**
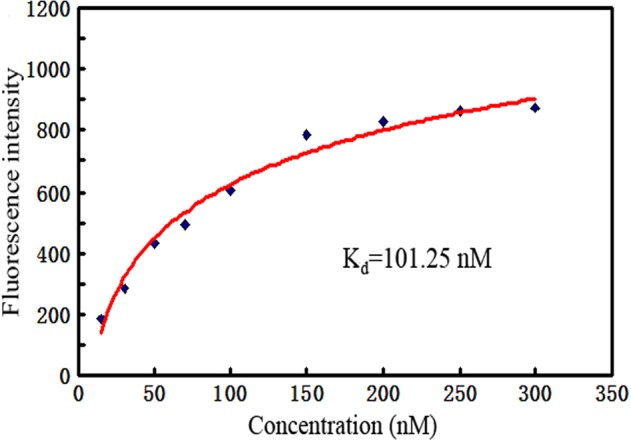
Evaluation of the aptamer’s binding affinity to OFA/iLRP. FAM-labeled aptamer AB3 of various concentrations were incubated with OFA/iLRP peptide-coated beads, which were analyzed for fluorescence intensity by flow cytometry. The K_d_ was calculated based on values of fluorescence intensity obtained at various concentrations of aptamer.

To quantitatively evaluate the binding affinity of AB3 to OFA/iLRP structure, the K_d_ value was estimated. Briefly, beads coated with OFA/iLRP-peptide were incubated with FAM-labeled AB3 of various concentrations, and evaluated for fluorescence intensity by flow cytometry. The K_d_ of the aptamer for binding with the OFA/iLRP-peptide was calculated to be 101.25 nM via non-linear regression analysis (Fig. 3).

### Aptamer AB3 selectively bound to OFA/iLRP-expressing tumor cells

**Figure 4:**
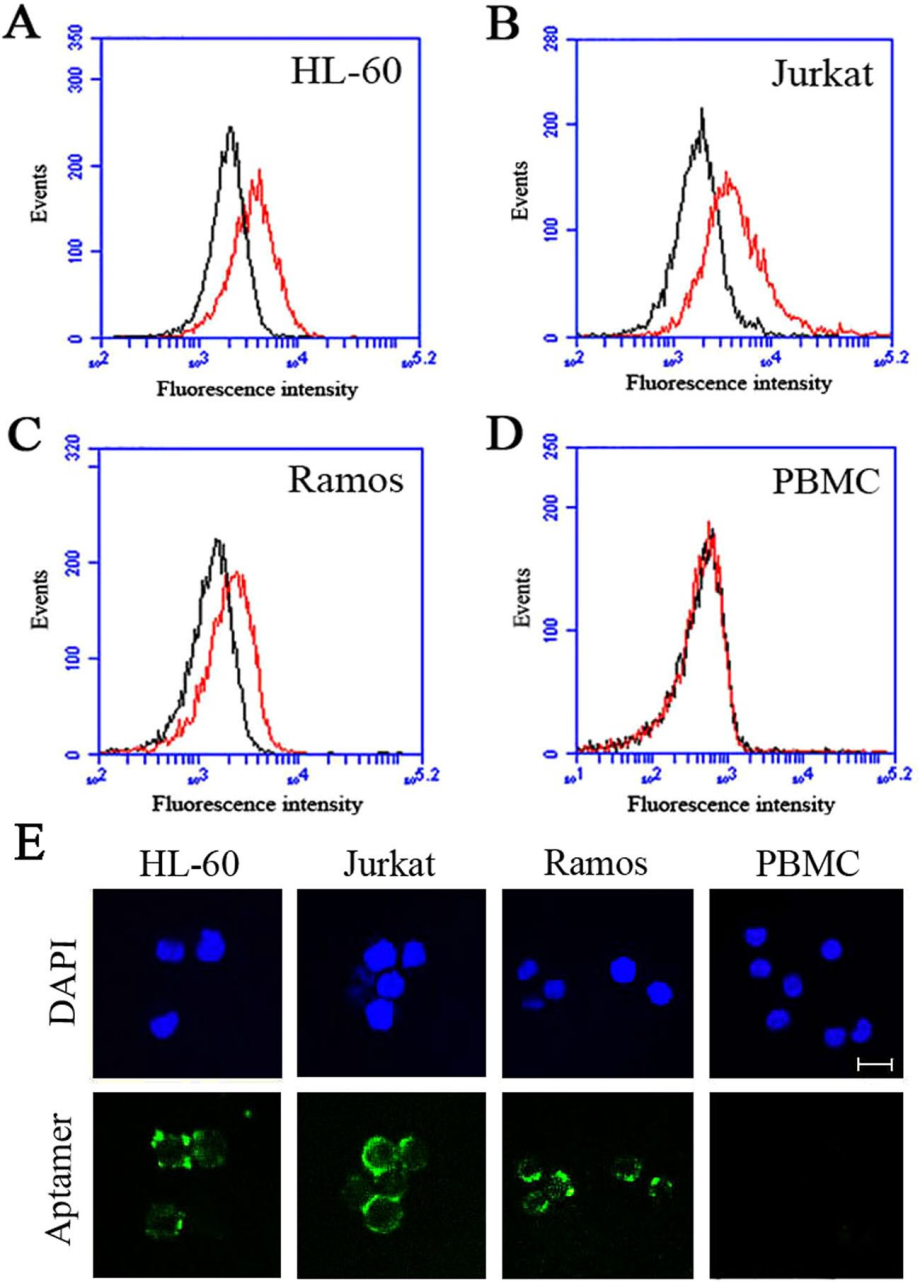
The bindings of aptamer AB3 to OFA/iLRP-positive and -negative cells. FAM-labeled AB3 were incubated with OFA/iLRP-positive cells HL-60 (**A**), Jurkat (**B**), or Ramos (**C**), and OFA/iLRP-negative cells PBMC (**D**). The cells were washed and analyzed by flow cytometry. The red curves represented the fluorescent signals of the aptamer and the black curves represented the control fluorescence signals generated by FAM-labeled initial ssDNA library. (**E**) Confocal microscopy evaluation of the aptamer’s binding to OFA/iLRP-positive and -negative cells. Green fluorescence signals were generated by FAM-labeled aptamer. The nuclei were stained blue with DAPI. Bar represents 10 μm.

Although aptamer AB3 had certain binding affinity and specificity for OFA/iLRP-peptide, it was unknown whether the aptamer could also bind to OFA/iLRP-expressing cancer cells. To address this issue, OFA/iLRP-positive cells (HL-60, Jurkat, and Ramos) and OFA/iLRP-negative control cells (PBMC) were incubated separately with FAM-labeled aptamer, washed, and analyzed by flow cytometry. As presented in Fig. 4, the aptamer AB3 generated strong bindings to OFA/iLRP-positive cells (Fig. 4A–C), and bound weakly to OFA/iLRP-negative control cells (Fig. 4D). The results indicated that the AB3 aptamer could recognize and bind with the OFA/iLRP-positive cancer cells.

To further investigate the binding preference of aptamer AB3 to OFA/iLRP-positive and -negative cells, confocal microscopy were also used to study HL-60, Jurkat, Ramos, and PBMC cells treated by FAM-labeled aptamers. As shown in Fig. 4E, the fluorescence signal was observed mainly on the surface of OFA/iLRP-positive cells but not on OFA/iLRP-negative control cells. The results suggested that the aptamer AB3 could selectively bind with OFA/iLRP-positive cells, presumably by recognizing the extracellular domain of OFA/iLRP on these cells.

### Aptamer AB3 targeted membrane proteins on the surface of OFA/iLRP-expressing cells

**Figure 5:**
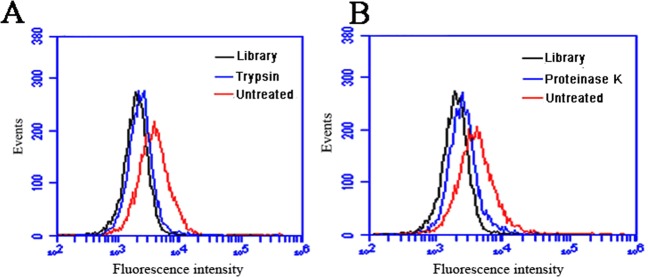
Effects of proteinase treatment on aptamer’s binding to target cells. HL-60 cells were treated for 5 min with 0.25% trypsin (**A**) or 0.1 mg/mL proteinase K (**B**), washed with PBS, incubated with FAM-labeled aptamers, and analyzed with flow cytometry (blue lines). Digested cells treated with initial ssDNA library were used as the negative control (black lines). Untreated HL-60 cells incubated with FAM-labeled AB3 were used as the positive control (red lines).

To further evaluate whether the aptamer targeted the extracellular domain of membrane proteins on OFA/iLRP-expressing cells, HL-60 cells were treated with trypsin or proteinase K for 5 min, incubated with FAM-labeled AB3, washed, and analyzed by flow cytometry. After trypsin or proteinase K treatment, the aptamer’s binding to HL-60 cells was nearly abolished (Fig. 5). Based on the fact that the extracellular domains of membrane proteins, but not the other components of plasma membrane such as lipids and saccharides, were digested by trypsin or proteinase K^49^, the flow cytometry results suggested that the aptamer probably targeted the extracellular domain of OFA/iLRP on the surface of cancer cells.

### Formation of the aptamer-doxorubicin complex

**Figure 6:**
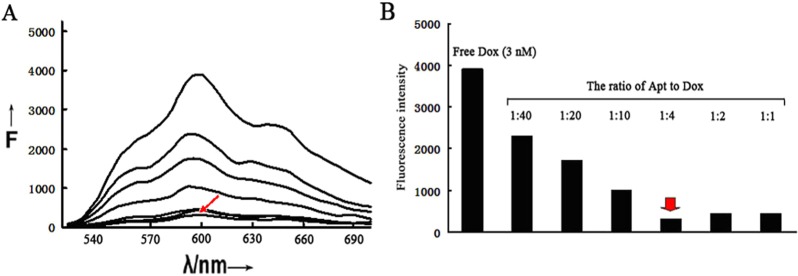
Fluorescence spectrum assessment of the aptamer-doxorubicin mixture. (**A**) Fluorescence spectrums of doxorubicin solution mixed with increasing molar ratios of the aptamer (From top to bottom: 0, 0.025, 0.05, 0.1, 0.25, 0.5 and 1). (**B**) Fluorescence intensities of doxorubicin solution mixed with increasing molar ratios of the aptamer at λEm = 600 nm. The red arrow indicated the point whereby most doxorubicin molecules were loaded in the aptamers.

Doxorubicin is one of the most extensively used drugs in chemotherapy and capable of inhibiting the proliferation of a wide variety of cancer cells. One of the acting mechanisms is that doxorubicin tends to intercalate into the DNA structures and interfere with the cellular functions^50^. This mechanism can also be used to construct aptamer-doxorubicin complex, in that the drug can be readily inserted into the DNA structure of an aptamer to form a relatively stable complex. To study whether doxorubicin could intercalate into aptamer AB3, a fixed concentration of doxorubicin was mixed with increasing concentrations of AB3 at various ratios. Fluorescence spectroscopy was applied to monitor the mixtures, based on the fact that the red fluorescence of free doxorubicin will be quenched when the drug incorporates with DNA^51^. As shown in Fig. 6, with increasing aptamer/doxorubicin ratio, the fluorescence intensity of the mixture gradually decreased. When the Apt/Dox molar ratio increased to 0.25, the fluorescence largely reached the lowest level, indicating that, at this ratio, most doxorubicin had intercalated into the DNA structure of aptamer AB3, and that aptamer-doxorubicin complex (Apt-Dox) was formed.

### Aptamer AB3 selectively delivered doxorubicin to OFA/iLRP-positive AML cells

**Figure 7:**
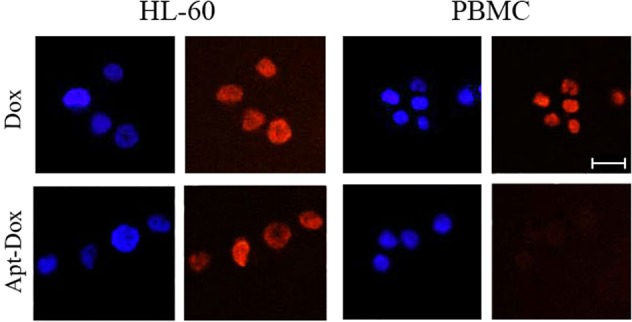
Cellular uptake of doxorubicin by OFA/iLRP-positive and -negative cells treated with free Dox or Apt-Dox. The OFA/iLRP-positive HL-60 cells and OFA/iLRP-negative PBMC were treated with free DOX (upper panel) or Apt-Dox (lower panel) separately for 2 h in PBS, and evaluated by confocal microscopy. The red fluorescence was generated by doxorubicin. The nuclei were stained blue with DAPI. Bar was 10 μm.

With the formation of Apt-Dox complex, we next investigated whether the aptamer could selectively deliver Dox to OFA/iLRP-positive AML cells. Drug uptake studies were performed *in vitro*. Specifically, OFA/iLRP-positive AML cells (HL-60) and OFA/iLRP-negative control cells (PBMC) were incubated with free Dox or Apt-Dox separately and evaluated by confocal microscopy. As shown in Fig. 7, when treated with free Dox, both HL-60 and PBMC cells exhibited strong red fluorescence (Fig. 7, the upper panel), indicating that free Dox non-selectively entered both types of cells. When treated with Apt-Dox, however, the red fluorescence in HL-60 was much stronger than that in PBMC (Fig. 7, the bottom panel), indicating that Dox mostly entered HL-60 but not the control cells. Taken together, the data demonstrated that free Dox non-selectively diffused into most cells, while Apt-Dox selectively delivered Dox into OFA/iLRP-positive AML cells.

### Apt-Dox generated a targeted cytotoxicity against the OFA/iLRP-positive AML cells

**Figure 8:**
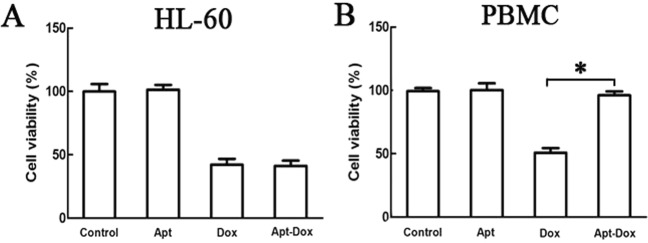
Cytotoxicity against OFA/iLRP-positive and -negative cells treated with Apt, Dox, or Apt-Dox. OFA/iLRP-positive cells HL-60 (**A**) and OFA/iLRP-negative cells PBMC (**B**) were incubated with aptamer, free Dox, or Apt-Dox for 4 hours, washed with PBS, and cultured for another 48 hours. MTS assay was employed to evaluate the cell viability (mean ± SD, n = 6). The star indicated a statistically significant difference between the group treated with free Dox and that with Apt-Dox (p < 0.01).

The above experiments indicated that Apt-Dox selectively delivered doxorubicin to OFA/iLRP-positive cells. We hypothesized that Apt-Dox would also generate a targeted cytotoxicity against the OFA/iLRP-positive leukemic cells. To test this postulate, HL-60 and PBMC cells were treated with free aptamer, free Dox, or Apt-Dox separately, and MTS assay was performed to analyze the cytotoxic effects of various treatments *in vitro*. As shown in Fig. 8, free Dox killed both the OFA/iLRP-positive and the OFA/iLRP-negative cells. By contrast, Apt-Dox largely destroyed the OFA/iLRP-positive HL-60 cells, but significantly reduced the damage to OFA/iLRP-negative control cells. Moreover, aptamer *per se* had no obvious cytotoxicity to either OFA/iLRP-positive or OFA/iLRP-negative cells, indicating that the aptamer was relatively nontoxic to both cell types. The results indicated that Apt-Dox generated a targeted cytotoxicity against the OFA/iLRP-positive AML cells, and simultaneously reduced the damage to OFA/iLRP-negative control cells.

## Discussion

In this study, we selected the first aptamer for recognizing OFA/iLRP, which is a potential therapeutic target because it is over-expressed on the surface of AML cells but under-expressed in normal tissue. The aptamer AB3 could bind to OFA/iLRP-epitope with a K_d_ of 101.25 nM, and had minimal cross-reactivity to BSA, OVA, or trypsin (Figs 2, 3). Moreover, the aptamer bound strongly to OFA/iLRP-positive AML/lymphoma cells, but weakly to OFA/iLRP-negative control cells (Fig. 4). By intercalating doxorubicin into the DNA structure of AB3, an aptamer-doxorubicin complex (Apt-Dox) was constructed (Fig. 6). Confocal microscopy revealed that Apt-Dox could selectively deliver doxorubicin into AML cells (HL-60), while significantly reduced the drug intake by OFA/iLRP-negative control cells *in vitro* (Fig. 7). Moreover, cytotoxicity study showed that Apt-Dox generated robust killing of AML cells, but mitigated the damage to OFA/iLRP-negative control cells (Fig. 8). These results indicate that the OFA/iLRP aptamer may potentially serve as a tumor-homing ligand in targeted therapy against AML.

Targeting OFA/iLRP for drug delivery represents a new strategy for AML treatment. Currently, the mainstream treatment for most AML is still chemotherapy with cytotoxic agents. Although chemotherapeutics can suppress AML cells, damages to normal hematopoietic stem cells are common and associated with severe side effects. Targeted tumor therapy may potentially reduce the side effects, because cytotoxic agents are selectively delivered to tumor cells. For targeted AML treatment, an antibody-drug conjugate (gemtuzumab ozogamicin) has been developed, aiming at delivery of cytotoxic agents to CD33-expressing AML cells. However, since CD33 is also expressed on normal myeloid cells, gemtuzumab ozogamicin has been reported to cause severe side effects at times, limiting its clinical applications^52^. Therefore, new AML-targeting strategy is still warranted at present. OFA/iLRP is highly expressed on most AML cells but under-expressed in normal myeloid cells, and thus may potentially serve as a molecular target for selective drug delivery to AML. In this study, the OFA/iLRP aptamer selectively delivered doxorubicin to HL-60 AML cells, and reduced the drug intake by control cells *in vitro* (Figs 7, 8). Although this new strategy for AML treatment still needs further development, the results suggest that targeting OFA/iLRP for delivery of cytotoxic agents is theoretically feasible.

The mechanisms by which free doxorubicin or Apt-Dox enter the OFA/iLRP-positive AML cells and the control cells were probably different. Based on previous studies^53^, we proposed the following hypothesis. For free doxorubicin, its lipophilic nature enabled the drug to diffuse into both types of cells with no selectivity. For Apt-Dox, doxorubicin was inserted within the DNA structure of aptamer and thus could not freely diffuse into cells. When OFA/iLRP-negative control cells were treated with Apt-Dox, the complex could not enter the cells, because both Apt-Dox and cell membrane were negatively charged and were repulsive to each other. When OFA/iLRP-positive AML cells were treated with Apt-Dox, however, the binding of the aptamer to OFA/iLRP on these cells could overcome the repulsive force and attach Apt-Dox to cell membrane. This led to the endocytosis of the Apt-Dox complex and the intake of doxorubicin by AML cells. Although this hypothesis may partially explain the targeting preference of Apt-Dox, further studies are warranted to reveal the detailed mechanism by which Apt-Dox selectively entered OFA/iLRP-positive AML cells.

It should be noted that, in addition to AML, OFA/iLRP is also widely expressed in other malignancies, and thus may potentially serve as a therapeutic target for a broader range of cancers. Holtl *et al*. reported that in an immunotherapy trial of renal cell carcinoma (RCC), OFA/iLRP-pulsed DC-based vaccine was well tolerated and had immunological as well as clinical effects in patients with metastatic RCC. The results indicated that OFA/iLRP might be an attractive candidate of tumor antigen for DC-based immunotherapy of RCC^54^. Siegel *et al*. reported that a powerful immune reaction against B-cell leukemia could be induced in mice immunized with DCs transfected with the mRNA of OFA/iLRP, indicating that B-cell leukemia could be treated with immunotherapies based on OFA/iLRP^11^. In this study, we also observed that the OFA/iLRP aptamer could bind to other types of tumor cells in addition to AML cells. Specifically, in addition to the AML cell line (HL-60), the aptamer was capable of recognizing T lymphocytic leukemia cells (Jurkat) and B-cell lymphoma cells (Ramos) as well (Fig. 4), consistent with prior studies demonstrating that Jurkat and Ramos cells were OFA/iLRP-positive^11^. These findings suggest that, in addition to AML, targeting OFA/iLRP may also have potential for treatment of other malignancies.

In this study, an OFA/iLRP-binding aptamer was selected as the tumor-homing ligand. Although most targeted drug delivery systems employ antibodies as the tumor-homing ligand, aptamers also have great application potential in drug development as a new class of ligands. The US FDA has approved Macugen as the first aptamer drug for treatment of age-related macular degeneration^55^. At present, there are several aptamer-based therapeutics that are under clinical trials of various phase^56,57^. Aptamers have certain advantages of potential drug candidates or tumor-targeting ligands, including high affinity, excellent specificity, low immunogenicity, easy chemical modification, and low production cost. In order for aptamers to be utilized *in vivo*, they usually need to be chemically modified to resist digestion by nucleases. Therefore, future studies should focus on structural modifications of the OFA/iLRP aptamer, in order to improve its half-life for *in vivo* application. Future research should also address whether Apt-Dox still has the tumor-targeting anticancer effects *in vivo* with animal studies. Furthermore, the OFA/iLRP aptamer may also conjugate with drug-carrying nanoparticles to construct novel targeted drug delivery systems for AML treatment.

In conclusion, OFA/iLRP is widely expressed in AML and other types of malignancies, and may serve as a potential therapeutic target. In this study, we selected the first aptamer against OFA/iLRP, which may serve as a tumor-homing ligand for targeted therapy against OFA/iLRP-expressing tumors.

## Methods

### Cells and cultures

The OFA/iLRP-positive cell lines, HL-60 (human acute promyelocytic leukemia), Jurkat (human acute T lymphocyte leukemia), and Ramos (Burkitt’s Lymphoma) were obtained from the Cell Center of Chinese Academy of Medical Sciences (Beijing, China). Peripheral Blood Mononuclear Cells (PBMC) were isolated from healthy donors. All cell lines were incubated in RPMI-1640 medium (Gibco) supplemented with 10% fetal bovine serum (FBS), 100 U/mL penicillin and 100 ug/mL streptomycin. Cells were grown in a humid atmosphere with 5% CO_2_ at 37 °C. All donors were required to sign an informed consent. The protocol was approved by the Ethics Committee of Chinese Academy of Medical Sciences and Peking Union Medical College, and all methods were conducted in accordance with the Declaration of Helsinki.

### Isolation of PBMC

Blood was collected from healthy donors in vacutainer containing sodium heparin. Five mL of blood was transferred into a 15 mL centrifuge tube (NEST) and diluted with equal volume of 0.9% sodium chloride. The diluted blood was aliquoted into two 15 mL centrifuge tubes containing 5 mL lymphocyte separation medium (Tbdscicece, China) and centrifuged at 1500 rpm/min for 20 min. Buffy coat containing PBMC was collected in 50 mL centrifuge tube and washed twice with 0.9% sodium chloride by centrifugation at 1500 rpm/min for 10 min. After discarding the supernatant, PBMC was resuspended with RPMI-1640 containing 10% FBS, 100 U/mL penicillin and 100 ug/mL streptomycin.

### Reagents

Peptides of at least 95% purity were synthesized by Bootech (Shanghai, China). Bovine serum albumin (BSA) was purchased from Tbdscicece (Tianjin, China). Trypsin was purchased from Amresco (US). Ovalbumin (OVA) was purchased from Sigma (US). Oligonucleotide primers were synthesized by Invitrogen (Shanghai, China). Streptavidin-coated magnetic beads were purchased from Promega (US). Affimag UF magnetic microspheres (7−8 um) were purchased from BaseLine ChromTech (Tianjin, China).

### Immobilization of targets on magnetic beads

A peptide with the sequence of NQIQAAFREPR from the extracellular domain of OFA/iLRP was employed as the target for aptamer selection. The OFA/iLRP peptide was immobilized to epoxy magnetic beads by cross-linking of epoxy and amine groups. The epoxy magnetic beads (1 × 10^5^) were washed thrice with 500 uL of Phosphate Buffered Saline (PBS), resuspended in 200 uL of Carbonate Buffer solution (CBS, PH = 10.7) containing OFA/iLRP peptide (2 ug), and incubated at room temperature with gentle stirring for 12 h. After washing thrice with 500 ul of PBS, the beads were stored at 4 °C in PBS. The conjugation of beads with other reagents (BSA, OVA, or trypsin) was accomplished similarly.

### ssDNA library and primers

A 59-nt single strand DNA (ssDNA) library was used for aptamer selection. The ssDNA contained a central randomized sequence of 21-nt flanked by two 19-base sequences for primer annealing. The sequence was 5′-TGCGTGTGTAGTGTGTCTG(N21)CTCTTAGGGATTTGGGCGG-3′. A FAM-labeled forward primer, 5′-FAM-TGCGTGTGTAGTGTGTCTG-3′ was used to monitor the binding of ssDNA with targets during selection process. When necessary, a biotin-labeled reverse primer, 5′-biotin-CCGCCCAAATCCCTAAGAG-3′, was used in PCR for later separation of the two chains of double-strand DNA (dsDNA) into ssDNA.

### *In vitro* SELEX process

The initial ssDNA library (200 pmol) was dissolved in PBS, denatured at 95 °C for 5 min, and cooled immediately on ice for 15 min. The ssDNA was mixed with the OFA/iLRP peptide-coated magnetic beads in 200 uL PBS, which was shaked gently at room temperature for 50 min. The unbounded ssDNA was discarded by washing the beads thrice with PBS. The target-bound ssDNA was eluted from beads by heating at 95 °C for 5 min and collecting the supernatent. The harvested oligonucleotides were amplified by PCR with FAM-labeled forward primer and biotin-labeled reverse primer (95 °C for 5 min, 18–24 cycles of 30 s at 95 °C, 30 s at 56 °C, and 40 s at 72 °C, and 72 °C for 10 min). The streptavidin-coated magnetic beads were mixed with PCR product, shaken for 20 min at room temperature, and washed with PBS. The dsDNA was denatured in alkaline condition (0.1 M NaOH) for 5 min. The beads were magnetically separated and discarded, to separate the bead-bound biotin-labeled antisense ssDNA from the FAM-labeled ssDNA in supernatent. The FAM-labeled ssDNA would be used for cytometric analysis or the next round selection. After eight rounds of selection, the enriched target-binding ssDNA pool was amplified by PCR with unmodified primers, and cloned into Escherichia coli using the TA cloning kit (Transgen) for DNA sequencing.

### Flow cytometric analysis

OFA/iLRP peptide-, BSA-, OVA-, or trypsin-coated magnetic beads (2 × 10^5^) were incubated with 20–40 pmol FAM-labeled ssDNA in 200 uL PBS for 30 min at room temperature, washed once or twice with 200 uL PBS, resuspended in 200 uL PBS, and analyzed by flow cytometry (Accuri C6 Flow Cytometer, BD). Flow cytometry analysis of cells (2 × 10^5^) was conducted in the similar way, except that the amount of ssDNA used was 40–60 pmol. To estimate the equilibrium dissociation constant (K_d_) of the aptamer, magnetic beads coated with OFA/iLRP peptide were incubated with various concentrations of FAM-labeled aptamers and analyzed by flow cytometry. All experiments for binding assay were repeated for at least three times. The K_d_ was calculated by fitting the dependence of fluorescence intensity of specific binding on the concentration of the aptamers to the equation: Y = B max X/ (K_d_ + X).

### Confocal microscopic studies

HL-60, Jurkat, Ramos, or PBMC cells (2 × 10^5^) were washed thrice with PBS and incubated with 60 pmol FAM-labeled aptamers in 300 uL PBS for 30 min. After washing twice with PBS, the cells were fixed with 4% formaldehyde for 10 min at 4 °C. After washing cells twice again with PBS, cells were resuspended with 10 uL of DAPI with the concentration of 1 ug/ml for 5 min, mounted onto slide, and covered by a glass coverslip. The cells were imaged and analysed using a confocal fluorescence scanning microscopy (Perkin Elmer Ultraview, US).

### Effects of proteinase treatment on aptamer binding to leukemic cells

HL-60 cells (2 × 10^5^) were washed thrice with PBS and resuspended in 200 uL of PBS containing 0.25% trypsin or 0.1 mg/mL proteinase K at 37 °C for 5 min. FBS was added to inhibit proteinase activity before washing cells twice with PBS. The cells were incubated with 60 pmol FAM-labeled aptamer in 300 uL PBS for 30 min, washed with 200 uL PBS, suspended with 200 uL PBS, and analyzed by flow cytometry.

### Drug-loading capacity of the aptamer

The aptamer was heated at 95 °C for 5 min and cooled immediately on ice for 15 min. Next, a fixed concentration of Dox (3 nM) was incubated with the aptamer at varying aptamer/Dox molar ratios (0, 0.025, 0.05, 0.1, 0.25, 0.5, and 1) respectively for 1 h in a 96-well black plate. Synergy4 analyzer was used to read the plate for assessing the fluorescence spectrum (λEx = 488 nm, λEm = 520–700 nm).

### Cellular drug uptake studies

HL-60 or PBMC (3 × 10^5^) were incubated with either 450 pmol doxorubicin or 112.5 pmol Apt-Dox complex in 300 uL PBS for 2 h, washed twice with PBS, and fixed with 4% formaldehyde for 10 min at 4 °C. The cells were washed twice with PBS and mixed with 10 uL of DAPI (1 ug/ml) for 5 min. The cells were mounted onto slide, covered with a glass coverslip, and analyzed by confocal fluorescence scanning microscopy.

### *In vitro* cytotoxicity studies

HL-60 or PBMC cells (5 × 10^4^ cells per well) were grown in 96-well plates, treated with free doxorubicin at the final concentration of 0.2 µM, Apt-Dox complex at the final concentration of 0.05 µM, or aptamer at the final concentration of 0.05 µM at 37 °C for 4 h, washed thrice with PBS, and cultured for an additional 48 h. Cell viability was determined by MTS assay according to the standard protocol as outlined by the manufacturer (Promega, US).

### Statistical analysis

Statistical analysis was performed using the statistical SPSS 17.0 software. One-way ANOVA with Fisher’s least significant difference (LSD) post hoc comparisons at 99% confidence interval was used for statistical comparisons. All data are presented as mean and standard deviation (mean ± SD).
